# Untangling the Pea Root Rot Complex Reveals Microbial Markers for Plant Health

**DOI:** 10.3389/fpls.2021.737820

**Published:** 2021-10-12

**Authors:** Lukas Wille, Mario Kurmann, Monika M. Messmer, Bruno Studer, Pierre Hohmann

**Affiliations:** ^1^Department of Crop Sciences, Research Institute of Organic Agriculture (FiBL), Frick, Switzerland; ^2^Molecular Plant Breeding, Institute of Agricultural Sciences, ETH Zürich, Zurich, Switzerland

**Keywords:** arbuscular mycorrhizal fungi (AMF), pea root rot complex (PRRC), pea (*Pisum sativum* L), *Aphanomyces euteiches*, *Fusarium* spp., *Rhizoctonia solani*, grain legumes, plant-microbe interactions

## Abstract

Plant health is recognised as a key element to ensure global food security. While plant breeding has substantially improved crop resistance against individual pathogens, it showed limited success for diseases caused by the interaction of multiple pathogens such as root rot in pea (*Pisum sativum* L.). To untangle the causal agents of the pea root rot complex and determine the role of the plant genotype in shaping its own detrimental or beneficial microbiome, fungal and oomycete root rot pathogens, as well as previously identified beneficials, i.e., arbuscular mycorrhizal fungi (AMF) and *Clonostachys rosea*, were qPCR quantified in diseased roots of eight differently resistant pea genotypes grown in four agricultural soils under controlled conditions. We found that soil and pea genotype significantly determined the microbial compositions in diseased pea roots. Despite significant genotype x soil interactions and distinct soil-dependent pathogen complexes, our data revealed key microbial taxa that were associated with plant fitness. Our study indicates the potential of fungal and oomycete markers for plant health and serves as a precedent for other complex plant pathosystems. Such microbial markers can be used to complement plant phenotype- and genotype-based selection strategies to improve disease resistance in one of the world’s most important pulse crops of the world.

## Introduction

Breeding for disease resistance defends crops against plant pathogens and contributes to sustainable agriculture. Breeding disease-resistant crops face complex challenges at all scales, from molecular and cellular to the field and regional systems (Nelson et al., 2018). A paradigm shift is leading research toward understanding complex plant-microbe interactions beyond reductionistic experimental systems, which have shaped our current understanding of plant resistance. Today, plant diseases are being investigated more and more in the light of the pathobiome concept, i.e., where the effect of a disease agent is modulated by its microbial background and where disease aetiology, and finally plant resistance, is the result of co-occurring pathogens (Lamichhane and Venturi, 2015; Busby et al., 2016; Brader et al., 2017; Bass et al., 2019).

Plant-associated microbiota plays a key role in plant health, and the plant genotype, in turn, can shape the composition of plant-associated microbial communities (Berendsen et al., 2012). Two seminal experiments have shown that microbe-mediated plant resistance is highly heritable (Mark and Cassells, 1996; Smith et al., 1999). In a recent study, Wei et al. (2019) have shown that cotton cultivars differing in their resistance to verticillium wilt have distinct rhizosphere microbiome compositions. Similarly, the root endophyte composition of carrot (Abdelrazek et al., 2020) and lentil (Bazghaleh et al., 2020) cultivars could be related to the plant resistance levels. In addition, it was shown that plant domestication and resistance breeding are actively shaping the plant microbiome (Mendes et al., 2018b; Wagner et al., 2020). Together, these findings indicated a relation between host genotype, microbial composition, and disease resistance. In light of such findings, plant breeding offers the opportunity to harness positive plant-microbe interactions and strengthen crop resistance (Wei and Jousset, 2017; Hohmann et al., 2020).

Research is only at the beginning of understanding the factors that direct plants in the complex interactions with the associated microbiome and still little is known about how to steer beneficial associations between plants and microbes. Although challenging, it has been postulated to take this complexity into account early in the resistance breeding process (Wille et al., 2019; Oyserman et al., 2021). For the integration of microbiome information into plant breeding, it is crucial to identify key microbes that govern agronomically important traits.

Pea (*Pisum sativum* L.) is the most widely grown pulse in the temperate zones (Food and Agriculture Organization [FAO], 2019). Its cultivation is severely threatened by a plethora of soil-borne fungal and oomycete pathogens causing root and stem rots, which are *Aphanomyces euteiches*, *Didymella pinodes*, *Didymella pinodella*, *Fusarium avenaceum*, *Fusarium oxysporum*, *Fusarium redolens*, *Fusarium solani*, *Pythium* sp., and *Rhizoctonia solani* are among the most important causal agents of pea root rot (Kraft and Pfleger, 2001; Gaulin et al., 2007; Pflughöft et al., 2012; Alcala et al., 2016; Taheri et al., 2017). Control of these pathogens is difficult as they survive on plant debris or form resting structures in the soil, and it has been shown that increasing the frequency of pea or other legumes in the crop rotation provoke the build-up of root rot pathogens (Li et al., 2014; Bainard et al., 2017). Despite incremental progress in resistance breeding against individual pathogens, they remain a major constrain to pea cultivation (Infantino et al., 2006; Rubiales et al., 2015).

Although not well understood, there is evidence that different combinations of these pathogens interact synergistically and infect the plant conjointly, forming a pea root rot complex (PRRC) (Baćanović-Šišić et al., 2018; Chatterton et al., 2018). Co-infection of two or more microbial species can break down resistance against single pathogens and aggravate disease as shown for several multi-species pathosystems of pea (Kerr, 1963; Shehata et al., 1983; Peters and Grau, 2002; Willsey et al., 2018; Zitnick-Anderson et al., 2018). On the other hand, plant beneficial microbes were shown to be involved in the suppression of pathogens of the PRRC. For instance, the mycoparasite *Clonostachys rosea* can increase seed germination and reduce root rot in pea infected with different pathogens (Xue, 2003). Similarly, it has been shown that arbuscular mycorrhizal fungi (AMF) increase the resistance of pea against Aphanomyces root rot (Thygesen et al., 2004). Recently, both microbial taxa were found within a diverse fungal community that included several known pea pathogens (Wille et al., 2020). In line with that, the study of Xu et al. (2012a) has previously shown that the health status of pea is mainly related to the fungal community present in diseased roots but barely reflected by the fungal community in the soil.

Polymerase chain reaction assays targeting individual pathogens of the PRRC have been established and employed to study the presence, abundance, and synergistic or antagonistic interactions of selected pathogenic species. Through end-point PCR, the study of Chatterton et al. (2018) surveyed pea roots grown in Canadian fields over 4 years and confirmed the presence of the PRRC pathogens which are *A. euteiches*, *F. avenaceum*, *F. oxysporum*, *F. redolens*, and *F. solani*. Remarkably, *A. euteiches* could not be isolated from roots by means of traditional culturing methods, despite its frequent detection by PCR. Through qPCR, it was shown that *A. euteiches* facilitates root colonisation of *Fusarium* species eventually leading to increased root rot (Willsey et al., 2018). In conclusion, recent advances in qPCR assay developments of major PRRC pathogens and beneficial provided ample opportunities to unravel multipartite interactions within this pathobiome.

The main objective of this study was to identify microbial markers related to plant health. With a set of resistant and susceptible pea genotypes and four agricultural soils showing different levels of disease pressure, we assessed the soil- and genotype-dependent composition of selected pathogenic and beneficial fungi and oomycetes in diseased pea roots to link plant resistance to microbial abundances. By this, we aimed at identifying microbial key players in the PRRC and defining microbial markers for plant health.

## Materials and Methods

### Plant Growth and Phenotyping

The experiment involved eight peas (*P. sativum* L.) genotypes that were selected based on a previous study on root rot resistance. These genotypes showed contrasting levels of resistance to a PRRC present in naturally infested field soil (Kirchlindach) (Wille et al., 2020). The present selection includes four varieties and four genebank accessions from the USDA-ARS GRIN Pea Core Collection (Supplementary Table 1).

The eight pea genotypes were grown in soil collected from four agricultural field sites showing different levels of PRRC infestation, which were Soil from Feldbach (F; healthy), Kirchlindach (K; infested), Puch (P; infested), and Neu-Eichenberg (N; infested) (Supplementary Table 2). Sieved soil was stored in polypropylene boxes at 4°C in the dark until further use. For the control treatment, soils were sterilised (X-Ray irradiation 30–100 kGy, Synergy Health Däniken AG, Switzerland) and stored vacuum packed.

Pea seeds were surface-sterilised in 70% ethanol for 30 s followed by a 1:1 (v:v) ddH_2_O-bleach solution (M-Classic Javel Wasser, Migros, Switzerland; final concentration approx. 2.5%) for 10 min. Finally, seeds were thoroughly rinsed in ddH_2_O and soaked for 2 h. Seven seeds per genotype were planted in a 2:1 (v:v) mixture of soil and sterilised sand (Quartz d’Alsace, Kaltenhouse, France, 0.2–0.63 mm grain) in plastic pots (600 ml). Pots were arranged in a randomised complete block design with the factors “soil” (four levels) and “genotype” (eight levels) in four replications. Each experimental unit was set up as a pair of two pots containing untreated soil and sterilised soil, respectively. The four replications were sown on four consecutive days and harvested over 4 days in the same order. Plants were grown under controlled conditions in the growth chamber for 29 days. A 16/8 light/dark cycle was applied, providing a photosynthetically active photon flux density of 200 μmol m^–2^ s^–1^ over the waveband 400–700 nm. Plants were watered with tap water every 72 h by flooding the pots 4 cm high for 30 min. The growth chamber means temperature over the course of the experiment was 20°C, relative humidity 85%. Pots were inspected on a daily basis for seedling emergence and plants were thinned out to reach a maximum of five plants per pot.

The plants were removed from the pots 29 days after sowing, and roots were washed under running tap water. A root rot index [RRI; 1 = healthy; 6 = complete root rot, plant dead (Wille et al., 2020)] was attributed to individual plants. Roots were separated from shoots with clean scissors, and kept on ice before storage at –20°C. Shoots were dried at 105°C until constant weight before recording dry weight. Biomass measurements per pot were standardised with the number of plants per pot at harvest. Relative Shoot Dry Weight (SDW*_*Rel.*_*) was calculated by dividing the biomass of the untreated soil treatment by the biomass of the corresponding sterile control treatment of the same genotype in each replication.

### Quantification of Microbial Taxa in Diseased Pea Roots

Previously published qPCR assays were used to quantify ten microbial taxa in the roots of plants grown in the non-sterile treatment (Supplementary Table 3). Microbial taxa were selected based on information from previous studies, including a characterisation of the fungal community of diseased pea roots (Wille et al., 2020). As a control, roots of pea genotypes C1 and C2 grown in the sterilised soil were also analysed. Roots were lyophilised and then ground to a fine powder for 20 s at 25 Hz in a Mixer Mill (Retsch, Haan, Germany) using one 20 mm steel bead. DNA was extracted from ∼20 mg root powder using the Mag-Bind^®^ Plant DNA DS 96 Kit (Omega Bio-Tek, Norcross, United States) according to the instructions of the manufacturer. DNA concentrations were measured photospectrometrically and samples were normalised to a DNA concentration of 50 ng μl^–1^. DNA extractions and subsequent qPCR analyses were done on a per pot basis (roots of all plants in one pot pooled) in two technical replications. The average between both technical replicates was used for all statistical analyses.

Standard curves were obtained using 10-fold serial dilutions of target DNA (10^3^ to 10^0^ pg μl^–1^). To approximate the ratio between target DNA and plant DNA, the serial dilutions were established in diluted plant DNA (50 ng μl^–1^), extracted from axenically grown (X-ray sterilised sand, ultra-pure water) pea seedlings (cv. “Respect”). For each target microbial taxon, DNA was extracted in the same way as the plant material from a patch of mycelium (∼7 cm^2^) of 10-day-old cultures grown on potato dextrose agar in the dark at room temperature (isolates used in this study are listed in Supplementary Table 3). For the AMF assay, five standard curves were obtained using 10-fold serial dilutions (10^6^ to 10^2^ copies μl^–1^) of transformed plasmids containing an AMF 18S rDNA sequence. Two replicate reactions were run on a Rotor-Gene Q Thermocycler (QIAGEN, Hilden, Germany) for each of the two extracted DNA samples resulting in a total of four technical replicates per pot. The 13 μl qPCR reactions contained 1.5 μl of template DNA, 6.5 μl of KAPA FAST qPCR master mix (Roche, Basel, Switzerland), primers, and probe, where necessary. PCR programs consisted of an initial denaturation step for 5 min at 95°C, followed by 40 cycles of 10 s at 95°C, 30 s at the assay-specific temperature (Supplementary Table 2), and 10 s at 72°C.

### Statistical Analysis

Statistical analyses were performed with R 3.6.1 (R Core Team, 2018). The R Markdown file for the analyses is provided on *https://github.com/dendrologicus*. SDW*_*Rel.*_* was analysed using linear regression according to the model: Y ∼ soil + genotype + soil:genotype + replication, where the factor “soil” has four levels, “genotype” has eight levels, and “replication” has four levels. SDW*_*Rel.*_* was transformed using an inverse Lambert W × *F*_*X*_ function before analysis using the R package *LambertW* (Goerg, 2015). Compliance with the model assumptions was controlled by visual inspection of the residual plots. The significance of the factors was tested using ANOVA with type III calculation of the sums of squares. Pairwise differences between soil means and genotypic means and subsequently planned contrasts between the susceptible and resistant genotype groups within the soils were calculated and tested for significance using Tukey’s honestly significant difference at a 5% level of significance using the R package *emmeans* (Lenth, 2019). RRI data was rank-transformed and analysed with a reduced model without the factor replication using the R package *ARTool* (Kay and Wobbrock, 2019). Data on RRI is presented in the Supplementary Information (Supplementary Figures 2–4).

Non-metric multidimensional scaling (NMDS; two dimensions) of the Bray-Curtis distances between samples was used to explore structural similarities between the microbial composition of the four soils and eight genotypes. Permutational multivariate analysis of variance (PERMANOVA) was used to test differences in the microbial composition among the different factor levels, i.e., “soil,” “genotype,” and their interaction. To assess if the groups of resistant and susceptible genotypes have different microbial communities, the analysis was also performed with the factor “resistance level” (two levels) replacing the factor “genotype” in the model. The associations between the 10 qPCR variables and each NMDS ordination were determined by calculating the goodness-of-fit statistic *r*^2^. NMDS, PERMANOVA, and goodness-of-fit were performed with R package *vegan* (Oksanen et al., 2019).

Gaussian copula graphical models were applied to study co-occurrence patterns among the ten microbial taxa in diseased pea roots. This approach allows to model the effect of environmental factors, i.e., “soil” in the present case, and biotic factors, i.e., mediator species, on the co-occurrence of species and to represent conditional dependencies in networks (Blanchet et al., 2020). Generalised linear models with a negative binomial distribution function were fit using the R package *mvabund* (Wang et al., 2020). For the model across the three infested soils, a fixed intercept for each soil was set. Then, *ecoCopula* was used to fit graphical models, calculate partial correlations and prepare the network visualisation (Popovic et al., 2019).

Generalised additive models (GAM) were fit using the R package *mgcv* (Wood, 2011) to explore the relationship of SDW*_*Rel.*_* and quantities of microbial taxa in the roots: *SDW_*Rel.*_* ∼ *microbial taxon 1* + *taxon 2* + … + *taxon 10*. Starting from this full model, a stepwise backward selection procedure was used, where at each step of the selection procedure the variable with the highest *P*-value was dropped to produce a final model with only significant (*P* > 0.05) smooth terms retained. Partial *r*^2^ for each retained variable in the final model was estimated by calculating the difference in the overall *r*^2^ of the final model and the final model without the variable in question. GAMs were calculated for the three infested soils individually and the three soils together. In addition, Spearman correlation between *SDW*_*Rel.*_ and microbial quantities were calculated within the three infested soils. To test the hypothesis of different quantities of the ten microbial taxa between the groups of resistant and susceptible pea genotypes, Wilcoxon rank-sum test was calculated for the three soils together.

## Results

### Plant Phenotypic Assessments

**FIGURE 1:**
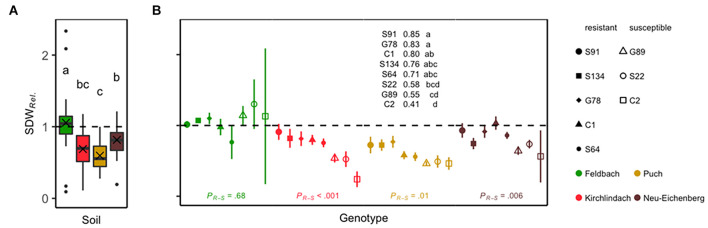
Relative shoot dry weight (SDW*_*Rel.*_*) of eight peas (*Pisum sativum*) genotypes grown for 29 days under controlled conditions in the four soils Feldbach, Kirchlindach, Puch, and Neu-Eichenberg. **(A)** Boxplots for each soil overall genotypes and replicates (*n* = 32) showing the median and the interquartile range; the ends of the whiskers represent 1.5 times the interquartile range; the mean is indicated by a cross. Soil means followed by a common letter are not significantly different (*P* > 0.05, Tukey HSD). **(B)** Mean SDW*_*Rel.*_* for eight pea genotypes (symbols) in each soil (colour): Solid symbols represent pea genotypes categorised as resistant; open symbols represent susceptible pea genotypes. Bars represent the SE of the mean. Genotypic means are presented over the three infested soils; means followed by a common letter are not significantly different (*P* > 0.05, Tukey HSD). Statistical significances of the difference between resistant and susceptible genotypes within each soil as tested by planned *post hoc* contrasts are indicated at the bottom.

In the K, P, and N soils, shoot dry weight in the non-sterile treatment was significantly reduced compared with the sterile treatment. No reduction in plant growth was observed in the healthy control soil F (Supplementary Figure 1). The factors “soil” and “genotype” had a significant effect on SDW*_*Rel*_* (*F*_3,86_ = 16.8, *P* < 0.001 and *F*_7,86_ = 4.2, *P* < 0.001, respectively). The interaction between “soil” and “genotype” was not significant (*F*_21,86_ = 1.11, *P* = 0.356). Mean (SD) SDW*_*Rel.*_* was 1.06 (0.43) in the F soil. SDW*_*Rel.*_* was significantly lower in the three infested soils (Figure 1A). In the F soil, susceptible and resistant genotypes did not have significantly different SDW*_*Rel.*_* [estimated difference (Tukey’s HSD) = 0.04, *P* = 0.68]. SDW*_*Rel.*_* was significantly lower for susceptible genotypes in the three infested soils (K = –0.38, *P* < 0.001; *P* = –0.20, *P* = 0.014; *N* = –0.25, *P* = 0.006). To analyse the growth performance of the pea genotypes on infested soil further, ANOVA was performed for the three infested soils. This revealed again significant effects of “soil” (*F*_2,65_ = 9.5, *P* < 0.001) and “genotype” (*F*_7,65_ = 8, *P* < 0.001). The interaction between these two factors was not significant (*F*_14,65_ = 1, *P* = 0.466). Therefore, *post hoc* analysis was calculated for genotypic means over the three infested soils, revealing significant differences for SDW*_*Rel.*_* between the pea genotypes, with genotype S91 being the most resistant and C2 the most susceptible (Figure 1B). The assessment of root rot symptoms (RRI) was in line with SDW*_*Rel.*_* data, but it differentiated poorly between the genotypes (Supplementary Figure 2).

### Quantification and Composition of Key Microbial Taxa

On all ten qPCR assays, average efficiencies and *R*^2^ of the standard curves were 1 (min. 0.97/max. 1) and 0.91 (0.71/1.17), respectively (Supplementary Table 4). In the control samples of pea roots (sterilised soil), 63 out of 320 tests resulted in DNA quantities above 1 pg rct^–1^ (median of 3 pg rct^–1^).

**FIGURE 2:**
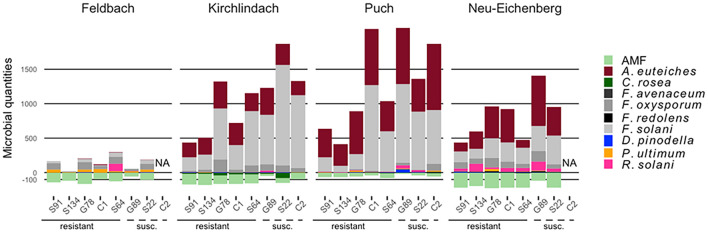
Composition of 10 microbial taxa in diseased pea (*P. sativum*) roots. Microbes were quantified by quantitative real-time PCR in roots of eight different pea genotypes grown in four different soils (Feldbach, Kirchlindach, Puch, and Neu-Eichenberg): Mean quantification [in pg rct^− 1^, or copies rct^− 1^ for arbuscular mycorrhizal fungi (AMF); *n* is given in Supplementary Table 5] of the 10 microbial taxa are given, with pathogens extending above of the 0-scale bar, beneficial taxa below (AMF quantifications were square-root transformed for this presentation). Pea genotypes are ordered based on relative shoot dry weight (high to low) in the initial resistance screening (Wille et al., 2020).

*Aphanomyces euteiches* and *F. solani* were the most abundant pathogens in diseased roots, distinguishing the healthy F soil from the three infested soils (Figure 2 and Supplementary Table 5). With a mean (*SD*) of 609 (324) pg rct^–1^, the diseased roots from P soil showed the highest *A. euteiches* concentrations compared with 359 (304), 299 (136), and 7 (13) pg rct^–1^ for the N, K, and F soil, respectively. DNA concentrations of *F. solani* were highest in the K soil [666 (578) pg rct^–1^] compared with the P [637 (674) pg rct^–1^], N [254 (273) pg rct^–1^], and F [44 (67) pg rct^–1^] soil. *F. oxysporum* and *R. solani* were quantified at intermediate levels: *F. oxysporum* was present in all soil-genotype combinations, however, considerably lower in the P soil [22 (35) pg rct^–1^] compared with the three other soils. *R. solani* showed considerably higher levels in the N soil [64 (77) pg rct^–1^] than the other soils. In the K soil, *F. oxysporum* was more present in roots from genotypes that had high overall pathogen loads (Figure 2). *D. pinodella*, *F. avenaceum*, *F. redolens*, and *P. ultimum* were detected at low levels (overall means < 13 pg rct^–1^). *F. avenaceum* was almost exclusively detected in samples grown in the P soil [3 (6) pg rct^–1^] and *F. redolens* in the N soil [7 (7) pg rct^–1^]. *P. ultimum*, on the other hand, showed the highest concentrations in the roots from the F soil [32 (33) pg rct^–1^].

There was a tendency that resistant pea genotypes had lower total pathogen amounts in the roots than susceptible genotypes (Figure 2). Genotypes S91 and S134 consistently showed low total pathogen abundance across the three infested soils. Genotypes S64 and G78 took an intermediate position in the P soil but showed pathogen amounts comparable to the susceptible genotypes in the K soil. While in the N soil, S64 showed total pathogen DNA levels as low as S91 and S134, wherein pathogen levels of G78 were as high as in the susceptible genotype S22. The resistant genotype C1 showed higher total pathogen concentrations than S91 and S134 with levels as high as susceptible genotypes in the P soil, which showed the highest disease pressure.

The potential fungal antagonist *C. rosea* was detected at low levels in samples from the F, P, and N soils (overall means < 10 pg rct^–1^) and at intermediate levels in samples from the K soil [29 (34) pg rct^–1^], generally uniformly present over all genotypes (Supplementary Table 5). AMF could be detected in all soil-genotype combinations, with the highest levels in samples grown in the N soil [41.1 (26.1) × 10^3^ copies rct^–1^].

**FIGURE 3:**
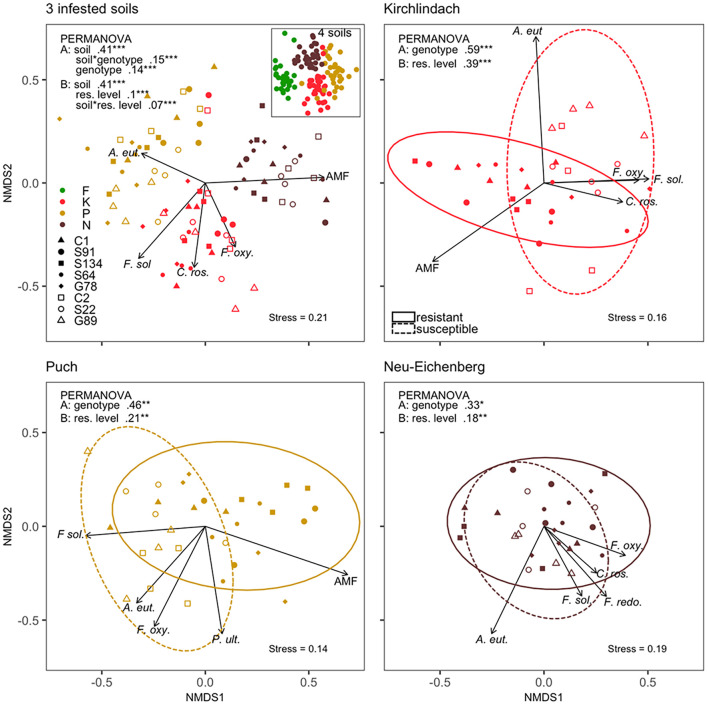
Composition of ten microbial taxa in diseased pea (*P. sativum*) roots. Microbes were quantified by quantitative real-time PCR in the roots of eight different pea genotypes grown in four different soils [Feldbach (F), Kirchlindach (K), Puch (P), and Neu-Eichenberg (N)]. Panels show the first two dimensions of the non-metric multidimensional scaling (NMDS) of the Bray-Curtis dissimilarities performed on quantities of the microbial taxa over three infested soils (inset: all four soils) or for each of the three infested soils individually. Arrows indicate the fitted microbial quantities; the arrow length is scaled by the respective *r*^2^ (goodness-of-fit with the ordination) of the variable. Only the top-five correlating variables are shown. Ellipses correspond to the 95% confidence interval of the factor “resistance level.” *R*^2^ and significance levels of the factors tested in the permutational multivariate analysis of variance (PERMANOVA) are provided: Analysis was performed with two models, either containing the factor “genotype” (A) or “resistance level” (B), and overall three infested soils and for each of the three infested soils individually (*P < 0.05; **P < 0.01; ***P < 0.001).

A clear clustering according to the soils was revealed by the non-metric multidimensional scaling (NMDS) of the composition of the ten microbial species between individual samples (Figure 3). Over the three infested soils, PERMANOVA indicated significant (*P* < 0.001) “soil,” “genotype,” and “soil × genotype” effects for the composition of ten microbial taxa in diseased pea roots, with 41, 14, and 15% of the variance in the microbial composition explained, respectively. When running the PERMANOVA with the factor “resistance level” instead of “genotype,” “resistance level” and the “soil × resistance level” interaction explained 10 and 7% of the variance, respectively. For each of the three infested soils, factors “genotype” or “resistance level” was significant, with the factor “genotype” (“resistance level”) explaining 59 (39), 46 (21), and 33% (18%) of the variance for K, P, and N, respectively. *A. euteiches*, *F. solani*, and *F. oxysporum* appeared as the main contributors of genotype separations with high (*r*^2^ > 0.3) and significant (*P* < 0.05) correlations with the first two dimensions of the ordinations of each of the three infested soils (Figure 3). AMF showed high and significant correlations in all but the N soil ordination, pointing toward the group of resistant genotypes.

**FIGURE 4:**
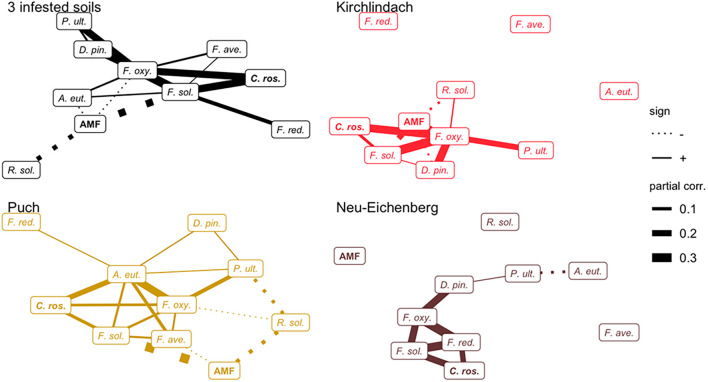
Networks of conditional dependencies among ten microbial taxa quantified in diseased pea (*P. sativum)* roots. Partial correlations between taxa were calculated using Gaussian Copula Graphical modelling across three infested soils and for each of the infested soils individually. Edge width is proportional to the strength of correlation; solid and dashed line types represent positive and negative correlations, respectively. Names of putative beneficial taxa (AMF and *C. rosea*) are in bold letters. *F. ave.*, *F. avenaceum*; *F. oxy*., *F. oxysporum*; *F. red.*, *F. redolens*; *F. sol.*, *F. solani*; *D. pin.*, *D. pinodella*; *R. sol.*, *R. solani*; *P. ult.*, *P. ultimum*; AMF, arbuscular mycorrhizal fungi; *C. ros.*, *C. rosea*.

Networks of conditional dependencies for each of the three infested soils individually showed that the majority of significant correlations between pathogenic species was positive (Figure 4). In each soil, *F. solani* and *F. oxysporum* consistently showed high connectivity (no. of edges). Additionally, AMF, *A. euteiches*, and *F. redolens* showed high connectivity in the K, P, and N soil, respectively. AMF showed negative associations with pathogenic species in K and P soils; the negative correlations between AMF and *F. solani* or *R. solani* were consistent across both soils. *C. rosea* showed positive associations with pathogenic taxa, consistently with *F. solani* in each soil. The network over the three infested soils corroborated the positive association between *F. solani* and *F. oxysporum*. These two species showed the highest connectivity (seven and six edges, respectively) among all taxa. AMF showed negative correlations with *A. euteiches*, *F. oxysporum*, *F. solani*, and *R. solani*.

### Relationship Between Plant Phenotype and Abundance of Microbial Taxa in Diseased Roots

**FIGURE 5:**
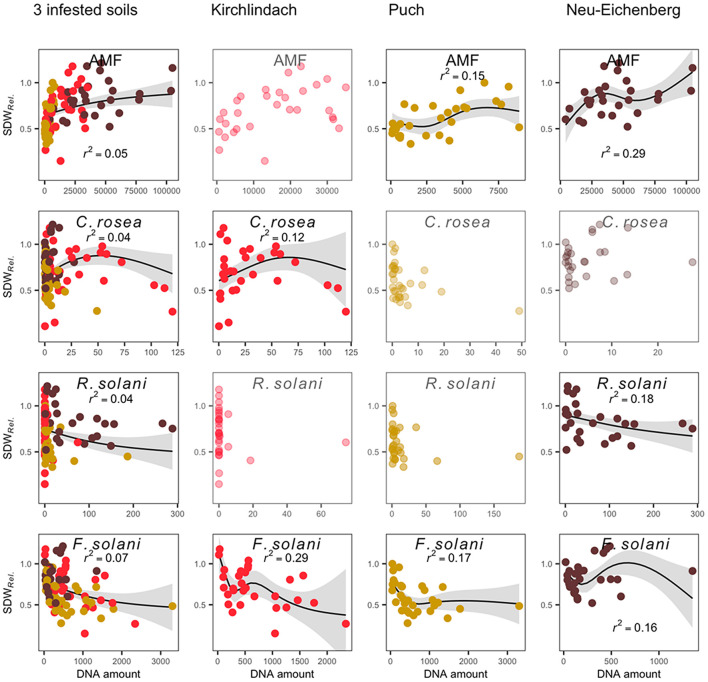
Generalised additive model (GAM) plots to show the relationship between qPCR-determined abundance of microbial taxa in diseased pea (*P. sativum*) roots and the relative shoot dry weight (SDW*_*Rel.*_*). Modelling was performed across three infested soils and for each soil individually. The relationship is only shown for significant smooth terms retained in the final model after stepwise backward selection (greyed out scatterplots are shown for microbial taxa retained in the three-soil model, but not the individual soil models). Original data (dots; each soil is represented by a distinct colour), GAM smooths (solid line with 95% confidence interval in grey), and partial *r*^2^ for each smooth term are presented. Microbial quantities are given in pg rct^− 1^; except for AMF, where quantities are given in copies rct^− 1^.

Generalised additive modelling of the relation between SDW*_*Rel.*_* and the abundance of microbial taxa in the diseased roots revealed a distinct model for each of the three infested soils (Figure 5). Adjusted *R*^2^ for the final models after stepwise reduction of smoothing terms were 0.53 for the model over the three infested soils and 0.43, 0.57, and 0.47 in the K, P, and N soil, respectively. In all three infested soils, significant smoothing terms for *F. solani* were retained in the final model, with negative trends in the K and P soils. AMF smooth terms were retained in the P and N soils showing a positive trend with SDW*_*Rel.*_* in both soils. In the N soil, *R. solani* showed a near-linear negative relation with SDW*_*Rel.*_* In K soil, *C. rosea* showed a bell-shaped relation with SDW*_*Rel.*_* GAM across the three infested soils confirmed the smooth terms retained in the individual soils, with negative trends for *F. solani* and *R. solani*, a positive trend for AMF, and a bell-shaped curve for *C. rosea*.

Analysis of Spearman correlations between SDW*_*Rel.*_* and microbial quantities confirmed the GAM analysis with consistently high correlations between AMF abundance and SDW*_*Rel.*_* in all three infested soils (Supplementary Figure 3). Furthermore, SDW*_*Rel.*_* showed negative correlations with *A. euteiches*, *C. rosea*, *D. pinodella*, *F. avenaceum*, *F. solani*, *F. oxysporum*, and *R. solani* in at least one of the three soils. Looking at the three soils together, the group of susceptible pea genotypes had significantly higher quantities of *A. euteiches*, *F. avenaceum*, *F. oxysporum* and *F. solani* (Supplementary Figure 4). AMF was more abundant in the roots of resistant genotypes.

## Discussion

This is the first report that conjointly characterised eight major pathogens of the PRRC and two beneficial fungal taxa assessing eight pea genotypes with contrasting field-relevant resistance capacities in different agricultural soils. Our study demonstrated the relation between plant genotype, plant resistance, and composition of key microbial taxa within a pathogen complex. This builds on other studies on legume pathobiomes that used metabarcoding (Xu et al., 2012a,b; Mendes et al., 2018a). Through deploying qPCR, we were able to specifically assess known key microbes of a pathogen complex. The present experiment confirmed the previously reported high PRRC disease pressure of the K soil with a mean shoot biomass reduction of 32% (Wille et al., 2020). Overall, the three infested agricultural soils produced SDW*_*Rel.*_* values in the range of previously reported data on pea root rot (Pilet-Nayel et al., 2005; Šišić et al., 2018). Plant fitness levels of the pea genotypes were stable across the infested soils, despite distinct pathogen compositions in the roots grown in these soils. Different pathogen levels recorded among genotypes with comparable plant fitness (SDW*_*Rel.*_*) reveals tolerance, i.e., enduring infection (Pagan and Garcia-Arenal, 2018), and resistance, i.e., low pathogen load, as two different host strategies to grow well in infested soils.

The assessed microbial taxa formed complex co-occurrence networks in each infested soil with several central taxa conserved across the three soils. Soil-dependent factors have repeatedly been documented as important drivers of plant-associated microbial composition (Peiffer et al., 2013; Chemidlin Prevost-Boure et al., 2014; Xue et al., 2018; Hohmann et al., 2020). Our data is in line with this fundamental observation but also suggested that several taxa form a core of key players of the PRRC. This is especially notable in *A. euteiches*, *F. oxysporum*, *F. solani*, and AMF which showed the highest centrality across the infested soils investigated in this study. *F. solani and A. euteiches* dominated the pathogen composition in diseased roots in the three infested soils. To the best of our knowledge, our study is the first report on the presence of *A. euteiches* in pea roots grown in German and Swiss soils. Both pathogens are well-known members of the pea root rot complex, mutually facilitating plant infection and aggravating disease symptoms (Peters and Grau, 2002; Willsey et al., 2018). The study of Willsey et al. (2018) reported a significant disease reinforcement effect of *A. euteiches* in greenhouse co-inoculation experiments. The observed co-occurrence of both pathogens in the P soil, the soil with the strongest root rot development, and across all infested soils confirms their dependency in the pathogen complex. Combined with higher levels of both pathogens in susceptible pea genotypes, our data confirmed previous findings of the importance of both pathogens in the PRRC, with the selection procedure of the GAM modelling indicating *F. solani* abundance to be the preferred predictor of disease susceptibility. *R. solani* was also identified as a predictor of disease susceptibility when levels in roots exceed a certain threshold (about 50 pg rct^–1^). *R. solani* is frequent in pea fields in the US, Canada, or China (Yang et al., 2005; Mathew et al., 2012; Melzer et al., 2016). The pathogen is known to be mainly related to seedling disease but can facilitate further infections of other pathogens (Gossen et al., 2016; Chatterton et al., 2018). Our data did not confirm previous findings as *R. solani* consistently showed no or negative correlations with other pathogens in the network analysis indicating an involvement independent from other key players. Based on our findings and given their global importance, we suggest focusing on *F. solani and A. euteiches* in resistance breeding programmes, acknowledging that the role of *R. solani* and its potential dynamic interactions need further investigation.

Together with *F. solani*, other *Fusarium* species have repeatedly been confirmed as prevalent pathogens associated with pea root rot in North America and Europe (Feng et al., 2009; Pflughöft et al., 2012; Chittem et al., 2015). In our experiment, *F. oxysporum* showed strong connectivity and collinearity with other pathogenic taxa in the three infested soils. However, it did not appear as a significant predictor of disease susceptibility or resistance. Various strains including non-pathogenic forms of *F. oxysporum* are known to opportunistically co-infect a host or even antagonise other pathogens (Oyarzun et al., 1994; Xu et al., 2012a; Šišić et al., 2018). Earlier experimental work suggested that *F. oxysporum* may not be a primary factor of pea root rot suggesting its fellow-runner behaviour in the root rot complex (Kerr, 1963; Chittem et al., 2015), despite being frequently isolated from root-rot infected fields (Kraft, 1994; Chatterton et al., 2018). The low abundance levels of *F. avenaceum* and *F. redolens* confirmed recent findings on low aggressiveness of *F. redolens* (Willsey et al., 2018), but stood in contrast to studies that show high aggressiveness of *F. avenaceum* in pea (Pflughöft et al., 2012; Chittem et al., 2015; Šišić et al., 2018). Likewise, *D. pinodella* was detected at low levels in the roots from all three infested soils. We could not confirm its importance in the PRRC despite its reported importance in European cropping systems (Persson et al., 1997; Pflughöft et al., 2012; Xu et al., 2012b) and confirmed aggressiveness on pea in controlled experiments (Baćanović-Šišić et al., 2018). Similarly, no clear conclusions can be drawn on the role of *P. ultimum* even though *Pythium* spp. are common root rot pathogens provoking damping-off (Kerr, 1963; Muehlbauer and Kraft, 1973; Pflughöft et al., 2012; Alcala et al., 2016). Although our results pointed at subordinate roles of several low-abundant pathogens, they could all be detected in pea roots of at least one of the three infected soils. The context-dependency of microbial interactions remains challenging to untangle (Hohmann et al., 2020). Environmental factors, especially soil type and crop rotation history, need to be taken into consideration and related facilitative effects within the PRRC need further investigation.

Arbuscular mycorrhizal fungi quantities in the roots showed mostly negative relations with pathogenic taxa in the network analysis, most notably with *F. solani*, *A. euteiches*, *R. solani*, and *F. oxysporum*, and positive relations with plant growth. Our data indicated that AMF can be readily detected in the roots at the seedling stage and supported previous observations of their protective effects in pea at early growth stages (Thygesen et al., 2004). This builds upon the well-known disease mitigation by AMF under controlled conditions (Azcón-Aguilar and Barea, 1996; Slezack et al., 2000), or their relation to the health status of pea in the field (Xu et al., 2012a). Irrespective of the underlying causation, our results highlighted AMF as one of the key predictors of PRRC disease resistance across three different infested soils. In the light of the fact that AMF was shown to be effective in various other crop pathosystems (Diagne et al., 2020), they hold promise to be a universally important element for sustainable resistance breeding.

*Clonostachys rosea* quantities in diseased roots were positively correlated with the most important pathogens. Its smooth term suggests a dose-dependent relation with plant fitness. In biocontrol experiments, the strain AC941 is usually employed (Xue, 2003; Gimeno et al., 2019), and we did not know if the mycoparasitic lifestyle of this strain is extendable to the whole species or what factors determine the transition from commensalism to parasitism. It has been shown to also act as a legume pathogen (Afshari and Hemmati, 2017). *C. rosea* might thrive on other fungi present in and around the roots, thus co-occurring with them.

Our study showed that the host genotype plays a significant role in determining the composition of selected taxa of the pea pathobiome. Susceptible and resistant pea genotypes had different microbial compositions overall, with generally lower amounts of key pathogens and higher amounts of AMF in the roots of resistant genotypes. The proportion of the variance in the root microbial composition explained by the factor genotype is in the range of previously published values (Lundberg et al., 2012; Bulgarelli et al., 2015; Leff et al., 2017). This makes the plant-associated microbiome in general, and the monitoring of microbial key players in the PRRC in particular, an auxiliary tool in resistance breeding.

*Fusarium solani* and AMF, as well as to a less conclusive extent *A. euteiches*, *R. solani*, and *C. rosea*, were identified as microbial markers for plant health. The use of abundance information in plant selection could be realised either in high-throughput at early screening stages involving several genotype-soil combinations, or as complementary indicators in addition to classical disease phenotyping at later breeding stages. These findings are in line with a previous study that emphasises genotype selection for enhanced interactions with AMF for resistance breeding (Hohmann and Messmer, 2017). In combination with classical disease phenotyping, microbial markers showed the potential to distinguish between disease resistance and tolerance. In our study, we identified high and low pathogen loads of genotypes with similar tolerance levels (based on SDW*_*Rel.*_*). For instance, genotypes C1 and G78, while tolerant, showed medium to high overall pathogen levels. Since pea root rot is caused by microbial dysbiosis in the pea rhizosphere as a result of legume intensive crop rotations (Niu et al., 2018), there might be a substantially higher risk of such dysbiosis when cultivating C1 or G78. Whereas S91 and S134, with their overall low pathogen loads, presented resistant genotypes with a lower risk of such accumulations. Finally, linking the pathogen composition with growth performance at later stages of the development (and finally to yield) offered an additional selection criterion and could be an instrument to improve the performance prediction of genotypes in the field.

Repeated experimental evidence supports the importance of pathogen complexes for the pea (Kerr, 1963; Shehata et al., 1983; Oyarzun and Van Loon, 1989; Xue, 2003; Wille et al., 2020) and other plant pathosystems (Lamichhane and Venturi, 2015; Abdullah et al., 2017). Notably, interactions of pathogens have significant implications for disease aetiology (Kerr, 1963; Willsey et al., 2018), plant resistance (Kankanala et al., 2019), and disease management (Gossen et al., 2016; You and Barbetti, 2019). Our results demonstrated that the composition of key microbial taxa of the PRRC in diseased pea roots was determined by conjoint effects of the soil and plant genotype. Furthermore, the current study provided crucial insights toward the use of microbial markers for resistance breeding. While our study assessed a naturally occurring pathobiome under controlled conditions, it added to pathogen surveys in the field and studies on artificial inoculations of plant microbes. This congregation of plant science and agricultural research comes at a time when microbiome research claims to contribute to sustainable agriculture in the years to come (D’Hondt et al., 2021). A better understanding of the role of co-occurring pathogens in disease formation will eventually provide essential knowledge to tackle current and future challenges in resistance breeding.

## Data Availability Statement

The datasets presented in this study can be found in online repositories. The names of the repository/repositories and accession number(s) can be found in the article/Supplementary Material.

## Author Contributions

PH, MM, and BS conceived the study. LW, MK, MM, and PH designed the experiment, in consultation with BS. LW and MK conducted the experimental work. LW analysed the data. LW and PH wrote the manuscript with contributions of MM and BS. All authors read and approved the final manuscript.

## Conflict of Interest

The authors declare that the research was conducted in the absence of any commercial or financial relationships that could be construed as a potential conflict of interest.

## Publisher’s Note

All claims expressed in this article are solely those of the authors and do not necessarily represent those of their affiliated organizations, or those of the publisher, the editors and the reviewers. Any product that may be evaluated in this article, or claim that may be made by its manufacturer, is not guaranteed or endorsed by the publisher.
