# Supplementary material for: Untangling the Pea Root Rot Complex Reveals Microbial Markers for Plant Health
Source: Front Plant Sci. 2021 Oct 12;12:737820. doi: 10.3389/fpls.2021.737820 (PMC8545811; doi:10.3389/fpls.2021.737820)
Supplement: Supplementary file 1 [file Data_Sheet_1.docx]

**SUPPLEMENTAL MATERIALS**

**Fig. S1** Shoot dry weight of pea plants grown under controlled conditions in four different soils (Feldbach, Kirchlindach, Puch and Neu-Eichenberg), either sterilised (S) or non-sterilised (NS). Shoot dry weight recorded after 29 days was normalised to one plant per pot. Boxplots show the median and the interquartile range; the ends of the whiskers represent 1.5 times the interquartile range; the mean is indicated by a cross. T-tests were performed to test significant differences between the means of the treatment NS and S (n = 32).


**Fig. S2** Root rot index (RRI; 1 = healthy; 6 = complete root rot, plant dead) assessed on eight pea genotypes grown for 29 days under controlled conditions in the four different soils Feldbach, Kirchlindach, Puch and Neu-Eichenberg. The RRI described by Wille *et al.* (2020) was attributed to individual plants and medians were calculated from scores of individual plants for each pot. A) Violin plots for each soil over all genotypes and replicates (n = 32). Soil means followed by a common letter are not significantly different (*P* > .05, Tukey HSD). B) Mean RRI for eight pea genotypes (symbols) in each soil (colour): Solid symbols represent pea genotypes categorised as resistant; open symbols represent susceptible pea genotypes. Bars represent the standard error of the means.


**Fig. S3** Spearman correlations between root rot index (RRI) and relative shoot dry weight (SDW*_Rel._*) and the abundance of ten microbial taxa. Mean (eight pea genotypes, four replications) quantities for each microbial taxon within soils are indicated in square brackets (pg rct^-1^; except for AMF where quantities are given in 10^3^ copies rct^-1^). The heatmaps show significant (*P* < .05) positive (yellow) and negative (dark blue) Spearman’s *ρ*.
**
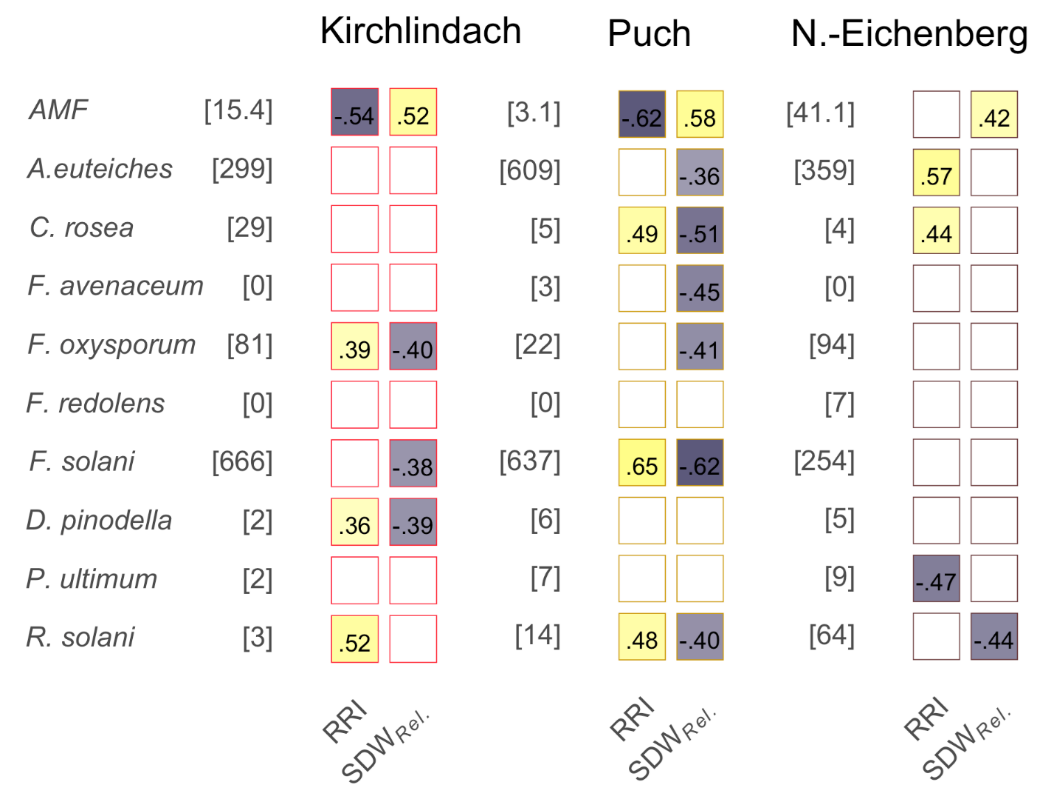
**

**Fig. S4** Comparison of root rot index (RRI) and relative shoot dry weight (SDW*_Rel._*) and abundances of ten microbial taxa in diseased pea roots between resistant and susceptible pea genotypes. Data was assessed on eight pea genotypes, categorized as 'resistant' (closed symbols) or 'susceptible' (open symbols) based on Wille et al. (2020), grown in three infested field soils (Kirchlindach, Puch and Neu-Eichenberg) under controlled conditions (four replications). Microbial taxa were quantified using quantitative real-time PCR; DNA concentrations are given in pg rct^-1^; except for AMF where quantities are given in copies rct^-1^. Boxplots show the median and the interquartile range; the ends of the whiskers represent 1.5 times the interquartile range. Wilcoxon rank sum tests were performed to test differences between the two resistance levels across the three sick soils; significance levels thereof are indicated in the plots (* *P* < .05; ** *P* < .01; *** *P* < .001; **** *P* < .0001).

**Table S1** Genebank accessions and cultivars of pea (*Pisum sativum*) evaluated for root rot resistance and microbial composition in diseased roots.

| ID | Cultivar name/ ARS-GRIN no. | Morphology: Leaf type, flower color, seed color, TGW [g] | Country of origin (breeding company)^a^ | PRRC resistance level^b^ |
| --- | --- | --- | --- | --- |
| C1 | E.F.B. 33 | Full leaf, pigmented, brown, 100 | DE (DSV) | R |
| S91 | PI 269777 | Full leaf, pigmented, 140 | Unilever, UK | R |
| S134 | PI 86430 | Full leaf, pigmented, green, 111 | Nepal | R |
| G78 | Roch | Full leaf, pigmented, brown, 180 | PL (PHR) | R |
| S64 | PI 241593 | Full leaf, pigmented, green, 200 | Taiwan | R |
| C2 | Respect | Semi leafless, white, yellow, 220 | FR(Agri Obtentions) | S |
| S22 | PI 164612 | Full leaf, white, green, 180 | India | S |
| G89 | Volt | Semi leafless, white, yellow, 250 | DE (NPZ) | S |

^a^DSV: Deutsche Saatveredelung; PHR: Poznaska Hodowla Roslin; NPZ: Norddeutsche Pflanzenzucht.

^b^Based on Wille et al. (2020); R = resistant; S = susceptible.

**Table S2** Soil characteristics of the four field soils used to assess the composition of root rot-associated microbes in diseased pea roots. For each soil 150 l were collected from the field, sieved using a 20 mm mesh and stored at 4°C in polypropylene boxes in the dark until further use. Representative samples were taken from the collected soil prior to the experiment. Grain composition, pH and soil nutrient analysis were performed by the Labor für Boden- und Umweltanalytik (lbu), Switzerland. N and C analysis were performed by the Research Institute for Organic Agriculture (FiBL), Switzerland according to (Agroscope, 1996).

|  | | |  |  | |  | | |  |  | | | |  |  | | | | | | | |
| --- | --- | --- | --- | --- | --- | --- | --- | --- | --- | --- | --- | --- | --- | --- | --- | --- | --- | --- | --- | --- | --- | --- |
|  |  |  | | | | Grain composition | | |  | Soil nutrient content [%] | | | |  | Soil nutrient content [mg kg^-1^] | | | | | | | |
| Field site (country) | | | Health | | Coordinates | Clay | Silt | Sand | pH | N*_tot_* | C*_tot_* | | C*_org_* |  | P | K | Mg | B | Mn | Cu | Fe |  |
| Feldbach (CH) - F | | | healthy | | 47.238882, 8.788594 | 28.3 | 34.0 | 37.7 | 7.1 | 0.27 | | 2.57 | 2.41 |  | 56.7 | 110.7 | 520.6 | 0.3 | 578 | 19.8 | 436 |  |
| Kirchlindach (CH) - K | | | infested | | 47.004760, 7.410004 | 16.5 | 37.4 | 46.1 | 7.1 | 0.19 | | 1.95 | 1.90 |  | 123.4 | 115.9 | 90.3 | 0.6 | 179 | 12.2 | 253 |  |
| Puch (DE) - P | | | infested | | 48.189986, 11.215125 | 17.9 | 51.0 | 31.1 | 6.8 | 0.34 | | 7.77 | 7.65 |  | 80.2 | 129.2 | 685.2 | 0.6 | 319 | 6.7 | 456 |  |
| Neu-Eichenberg (DE) - N | | | infested | | 51.379118, 9.910005 | 19.0 | 59.2 | 21.8 | 7.3 | 0.14 | | 1.45 | 1.44 |  | 63.1 | 130.5 | 148.1 | 0.6 | 383 | 6.8 | 316 |  |

**Table S3** Ten qPCR assays employed to detect microbial taxa in diseased pea roots. Indicated reference strains were used to prepare standard curves.

| Organism | Target | Reference strain | Primer/probe names | Primer/probe conc. [nM] | Annealing temp. | Reference |
| --- | --- | --- | --- | --- | --- | --- |
| AMF | 18s rRNA | Transformed plasmid | AMG1F/AM1 | 250/250 | 65.5°C | Hewins *et al.* (2015) |
| *Aphanomyces euteiches* | ITS I | AeRB84 (M.-L. Pilet; INRA Rennes, FR) | Ae_ITS1_39F/Ae_ITS1_167R | 250/250 | 61°C | Gangneux *et al.* (2014) |
| *Clonostachys rosea* | Actin gene | CRP1104 (A. ElHassan, Hohenh Uni., DE) | VTTact-f/VTTact-r/VTTact-pr | 300/300/100 | 62°C | Gimeno *et al.* (2019) |
| *Fusarium avenaceum* | EF-1α | F.ave_ku1 (A. Šišić, Kassel University, DE) | AveF/AveR/AvePr | 900/900/200 | 60°C | Zitnick-Anderson *et al.* (2018) |
| *Fusarium oxysporum* | ITS II | F.ox_ku1 (A. Šišić, Kassel University, DE) | FOF1/FOR1 | 300/300 | 65°C | Mishra *et al.* (2003) |
| *Fusarium redolens* | EF-1α | Isolate 1425 (A. Šišić, Kassel University, DE) | RedF/RedR/RedPR | 900/900/200 | 60°C | Zitnick-Anderson *et al.* (2018) |
| *Fusarium solani* | EF-1α | F.sol_ku1 (A. Šišić, Kassel University, DE) | SolF/SolR/SolPr | 900/900/200 | 62°C | Zitnick-Anderson *et al.* (2018) |
| *Didymella pinodella* | EF-1α | D.pin_ku1 (A. Šišić, Kassel University, DE) | Tef1F/Tef1R/Tef1Pr | 900/900/250 | 61°C | Šišić et al., in prep. |
| *Rhizoctonia solani* | ITS II | F122 (Geisenheim, DE) | ST-RS1/ITS4 | 300/300 | 60°C | Lievens *et al.* (2006) |
| *Pythium ultimum* | ITS II | 5808 (Syngenta, CH) | AFP276/ITS4 | 500/500 | 58°C | Lievens *et al.* (2006) |
|  |  |  |  |  |  |  |

**Table S4** Amplification efficiencies (E) and *R^2^* for the standard curves of ten qPCR assays targeting microbial taxa in diseased pea roots. Efficiencies and R2 are shown for every independent qPCR run. Quantifications were performed on two independent DNA extractions; "a" and "b", respectively. Each sample was tested in duplicate qPCR reactions. AMF = arbuscular mycorrhizal fungi.

| Biological rep. | | 1 | | 2 | | 3 | | 4 | | MEAN |
| --- | --- | --- | --- | --- | --- | --- | --- | --- | --- | --- |
| Technical rep. (DNA extraction) | | a | b | a | b | a | b | a | b |  |
|  |  |  |  |  |  |  |  |  |  |  |
| *A. euteiches* | *R^2^*: | 1.00 | 1.00 | 1.00 | 1.00 | 1.00 | 1.00 | 1.00 | 1.00 | 1.00 |
|  | E: | 0.86 | 0.84 | 0.81 | 0.84 | 0.72 | 0.85 | 0.82 | 0.86 | 0.83 |
| AMF | *R^2^*: | 1.00 | 0.99 | 0.99 | 0.99 | 0.99 | 1.00 | 0.98 | 0.99 | 0.99 |
|  | E: | 0.85 | 0.86 | 0.83 | 0.95 | 0.99 | 0.86 | 0.88 | 0.91 | 0.89 |
| *C. rosea* | *R^2^*: | 1.00 | 1.00 | 1.00 | 1.00 | 1.00 | 0.99 | 1.00 | 0.99 | 0.99 |
|  | E: | 0.99 | 0.94 | 0.98 | 0.93 | 1.05 | 0.99 | 1 | 0.93 | 0.98 |
| *D. pinodella* | *R^2^*: | 1.00 | 0.99 | 1.00 | 1.00 | 1.00 | 1.00 | 0.98 | 1.00 | 0.99 |
|  | E: | 0.71 | 0.79 | 0.83 | 0.78 | 0.77 | 0.87 | 0.81 | 0.83 | 0.80 |
| *F. avenaceum* | *R^2^*: | 0.99 | 0.99 | 0.98 | 0.99 | 0.97 | 1.00 | 0.99 | 0.99 | 0.99 |
|  | E: | 0.86 | 0.91 | 0.79 | 0.93 | 0.90 | 0.96 | 0.85 | 0.8 | 0.88 |
| *F. oxysporum* | *R^2^*: | 1.00 | 1.00 | 1.00 | 1.00 | 1.00 | 1.00 | 1.00 | 1.00 | 1.00 |
|  | E: | 0.87 | 0.90 | 0.84 | 0.88 | 0.97 | 0.85 | 0.88 | 0.92 | 0.89 |
| *F. redolens* | *R^2^*: | 1.00 | 1.00 | 1.00 | 1.00 | 1.00 | 1.00 | 1.00 | 0.99 | 1.00 |
|  | E: | 0.97 | 0.95 | 1.00 | 0.93 | 0.96 | 0.97 | 0.97 | 0.93 | 0.96 |
| *F. solani* | *R^2^*: | 1.00 | 0.99 | 0.99 | 1.00 | 1.00 | 1.00 | 1.00 | 1.00 | 1.00 |
|  | E: | 0.98 | 1.11 | 1.17 | 1.13 | 0.95 | 0.99 | 0.98 | 0.89 | 1.03 |
| *P. ultimum* | *R^2^*: | 1.00 | 1.00 | 1.00 | 1.00 | 1.00 | 1.00 | 0.99 | 1.00 | 1.00 |
|  | E: | 0.95 | 1.01 | 0.94 | 0.95 | 0.99 | 0.91 | 1.06 | 0.90 | 0.96 |
| *R. solani* | *R^2^*: | 1.00 | 1.00 | 1.00 | 1.00 | 1.00 | 1.00 | 1.00 | 1.00 | 1.00 |
|  | E: | 0.86 | 0.86 | 0.88 | 0.87 | 0.85 | 0.85 | 0.88 | 0.79 | 0.86 |
|  |  |  |  |  |  |  |  |  |  |  |

**Table S5** QPCR quantifications for ten microbial taxa in diseased roots of eight pea genotypes grown for 29 days in four different soils (F = Feldbach, K = Kirchlindach, P = Puch, N = Neu-Eichenberg). The mean ± SD of quantified microbial DNA (in pg rct^-1^) is presented along the number of analysed replicates (n; in brackets). DNA from each sample was independently extracted and subsequently tested twice; the average between both technical replicates was used for all statistical analysis. For better readability numbers were rounded to the nearest integer. For AMF, quantities are expressed as 103 ITS copies rct^-1^.

| **soil** | **Acc.** | ***F. ave.*** | ***F. oxy.*** | ***F. red*** | ***F. sol.*** | ***D. pin.*** | ***R. sol.*** | ***A. eut.*** | ***P. ult.*** | **AMF** | ***C. ros.*** |
| --- | --- | --- | --- | --- | --- | --- | --- | --- | --- | --- | --- |
| F | S91 | 0±0 (4) | 89±80 (4) | 0±0 (4) | 32±29 (4) | 0±0 (4) | 0±0 (4) | 0±0 (4) | 46±15 (4) | 19.1±6.5 (4) | 1±1 (4) |
|  | S134 | 0±0 (4) | 7±7 (4) | 0±0 (4) | 4±4 (4) | 0±0 (4) | 0±0 (4) | 1±1 (4) | 7±3 (4) | 14.4±5 (4) | 1±1 (4) |
|  | G78 | 0±0 (4) | 101±23 (4) | 0±0 (4) | 48±31 (4) | 3±3 (4) | 0±0 (4) | 11±18 (4) | 44±36 (4) | 27.4±7.1 (4) | 1±1 (4) |
|  | C1 | 0±0 (4) | 44±56 (4) | 0±0 (4) | 7±6 (4) | 0±0 (4) | 0±0 (4) | 18±18 (4) | 53±61 (4) | 9±3.2 (4) | 0±1 (4) |
|  | S64 | 3±5 (4) | 96±73 (4) | 0±0 (4) | 63±97 (4) | 0±0 (4) | 107±213 (4) | 13±23 (4) | 21±17 (4) | 16.4±6.9 (4) | 2±1 (4) |
|  | G89 | 0±0 (3) | 27±9 (3) | 0±0 (3) | 10±8 (3) | 0±0 (3) | 4±8 (3) | 0±0 (3) | 18±13 (3) | 2.9±1.3 (3) | 1±1 (3) |
|  | S22 | 0±0 (4) | 86±108 (4) | 0±0 (4) | 49±56 (4) | 5±7 (4) | 0±0 (4) | 5±8 (4) | 41±41 (4) | 13.1±8.7 (4) | 1±1 (4) |
|  | C2 | 0±0 (2) | 389±NA (1) | 0±NA (1) | 216±76 (2) | 25±33 (2) | 0±NA (1) | 0±0 (2) | 2±NA (1) | NA | 17±21 (2) |
|  | mean | 0±2 (29) | 77±89 (28) | 0±0 (28) | 44±67 (29) | 3±9 (29) | 16±81 (28) | 7±13 (29) | 32±33 (28) | 15.0±0.8 (25) | 2±6 (29) |
| K | S91 | 0±0 (4) | 23±22 (4) | 0±0 (4) | 191±166 (4) | 2±2 (4) | 1±3 (4) | 222±87 (4) | 1±1 (4) | 22.7±7.4 (4) | 21±22 (4) |
|  | S134 | 0±0 (4) | 41±30 (4) | 0±0 (4) | 220±232 (4) | 0±0 (4) | 0±0 (4) | 243±68 (4) | 1±1 (4) | 26.9±8.4 (4) | 15±12 (4) |
|  | G78 | 0±0 (4) | 180±206 (4) | 0±0 (4) | 741±483 (4) | 6±8 (4) | 1±3 (4) | 392±63 (4) | 0±0 (4) | 16.4±11.3 (4) | 43±46 (4) |
|  | C1 | 0±0 (4) | 42±31 (4) | 0±0 (4) | 352±202 (4) | 0±0 (4) | 0±0 (4) | 327±48 (4) | 0±0 (4) | 20.5±11.1 (4) | 24±23 (4) |
|  | S64 | 0±0 (4) | 98±49 (4) | 0±0 (4) | 783±532 (4) | 1±1 (4) | 0±0 (4) | 267±146 (4) | 6±7 (4) | 18.4±10.8 (4) | 25±11 (4) |
|  | G89 | 0±0 (4) | 92±44 (4) | 0±0 (4) | 717±511 (4) | 1±1 (4) | 23±35 (4) | 400±115 (4) | 1±1 (4) | 1.4±0.8 (4) | 16±27 (4) |
|  | S22 | 0±0 (4) | 99±44 (4) | 0±0 (4) | 1459±813 (4) | 1±0 (4) | 0±0 (4) | 310±73 (4) | 3±3 (4) | 5.1±3.4 (4) | 79±49 (4) |
|  | C2 | 0±0 (2) | 64±48 (2) | 0±0 (2) | 1059±16 (2) | 1±0 (2) | 0±0 (2) | 205±343 (3) | 1±1 (2) | 8.8±6 (2) | 3±5 (3) |
|  | mean | 0±0 (30) | 81±88 (30) | 0±0 (30) | 666±578 (30) | 2±3 (30) | 3±14 (30) | 299±136 (31) | 2±3 (29) | 15.4±11.3 (27) | 29±34 (31) |
| P | S91 | 1±1 (4) | 3±4 (4) | 0±0 (4) | 206±184 (4) | 0±0 (4) | 6±8 (4) | 418±140 (4) | 6±11 (4) | 4.3±1.9 (4) | 1±2 (4) |
|  | S134 | 0±0 (4) | 3±1 (4) | 0±0 (4) | 91±41 (4) | 1±0 (4) | 4±5 (4) | 315±75 (4) | 0±0 (4) | 4.4±2.6 (4) | 0±0 (4) |
|  | G78 | 1±1 (4) | 8±1 (4) | 1±1 (4) | 216±169 (4) | 3±4 (4) | 12±16 (4) | 629±337 (4) | 23±37 (4) | 4.3±4 (4) | 1±1 (4) |
|  | C1 | 1±0 (4) | 24±17 (4) | 0±0 (4) | 1238±1394 (4) | 0±0 (4) | 5±2 (4) | 818±278 (4) | 0±0 (4) | 1.4±1.5 (4) | 5±9 (4) |
|  | S64 | 0±0 (4) | 11±6 (4) | 0±0 (4) | 580±508 (4) | 2±3 (4) | 4±4 (4) | 439±200 (4) | 4±8 (4) | 5.3±2.5 (4) | 7±4 (4) |
|  | G89 | 9±11 (4) | 36±36 (4) | 0±0 (4) | 1147±458 (4) | 41±78 (4) | 49±92 (4) | 812±340 (4) | 6±7 (4) | 0.4±0.3 (4) | 4±5 (4) |
|  | S22 | 4±3 (4) | 14±11 (4) | 0±0 (4) | 833±558 (4) | 4±8 (4) | 24±29 (4) | 481±34 (4) | 0±0 (4) | 1.8±2.2 (4) | 2±2 (4) |
|  | C2 | 10±8 (4) | 98±71 (3) | 0±1 (3) | 781±344 (4) | 1±1 (4) | 1±1 (3) | 964±456 (4) | 17±24 (3) | 2.3±2.6 (2) | 14±23 (4) |
|  | mean | 3±6 (32) | 22±35 (31) | 0±0 (31) | 637±674 (32) | 6±28 (32) | 14±35 (31) | 609±324 (32) | 7±16 (31) | 3.1±2.7 (27) | 5±9 (32) |
| N | S91 | 0±0 (4) | 91±79 (4) | 4±3 (4) | 152±181 (4) | 10±14 (4) | 38±47 (4) | 135±67 (4) | 10±7 (4) | 46.6±10.3 (4) | 3±3 (4) |
|  | S134 | 0±0 (4) | 75±80 (4) | 4±3 (4) | 139±119 (4) | 7±13 (4) | 113±137 (4) | 261±184 (4) | 3±6 (4) | 38.2±15 (4) | 1±1 (4) |
|  | G78 | 0±0 (4) | 137±74 (4) | 12±7 (4) | 285±164 (4) | 5±5 (4) | 24±28 (4) | 464±297 (4) | 36±43 (4) | 53.2±36.9 (4) | 5±3 (4) |
|  | C1 | 0±1 (4) | 93±67 (4) | 6±6 (4) | 273±227 (4) | 3±3 (4) | 57±51 (4) | 490±210 (4) | 3±2 (4) | 48±20.8 (4) | 5±6 (4) |
|  | S64 | 0±0 (4) | 52±25 (4) | 4±6 (4) | 198±133 (4) | 2±2 (4) | 37±66 (4) | 104±47 (4) | 4±4 (4) | 34.3±32.3 (4) | 7±14 (4) |
|  | G89 | 1±0 (4) | 152±84 (4) | 14±13 (4) | 363±254 (4) | 7±4 (4) | 135±100 (4) | 734±216 (4) | 4±1 (4) | 14.7±7.5 (4) | 4±4 (4) |
|  | S22 | 0±0 (4) | 64±29 (4) | 4±3 (4) | 412±627 (4) | 2±1 (4) | 45±51 (4) | 416±451 (4) | 6±5 (4) | 53.3±40.3 (4) | 6±6 (4) |
|  | C2 | 0±NA (1) | 72±NA (1) | 1±NA (1) | 81±NA (1) | 0±NA (1) | 62±NA (1) | 0±NA (1) | 2±NA (1) | 41.1±NA (1) | 0±NA (1) |
|  | mean | 0±0 (29) | 94±67 (29) | 7±7 (29) | 254±273 (29) | 5±7 (29) | 64±77 (29) | 359±304 (29) | 9±18 (29) | 41.1±26.1 (29) | 4±6 (29) |
|  | mean | 1±3 (120) | 68±77  (118) | 2±5  (118) | 408±536  (120) | 4±15 (120) | 24±62  (118) | 325±316  (121) | 12±23  (118) | 18.6±20.4 (116) | 10±21  (121) |

*F. ave. = F. avenaceum; F. oxy. = F. oxysporum; F. red. = F. redolens; F. sol. = F. solani; D. pin. = D. pinodella; R. sol. = R. solani; P. ult. = P. ultimum;* AMF = arbuscular mycorrhizal fungi*; C. ros. = C. rosea.*
